# Rapid Screening of Active Components with an Osteoclastic Inhibitory Effect in *Herba epimedii* Using Quantitative Pattern–Activity Relationships Based on Joint-Action Models

**DOI:** 10.3390/molecules22101767

**Published:** 2017-10-19

**Authors:** Xiao-Yan Yuan, Meng Wang, Sheng Lei, Qian-Xu Yang, Yan-Qiu Liu

**Affiliations:** 1 Department of Analytical Chem istry of School of Pharmacy, Zunyi Medical University, Zunyi 563000, China; yuanxiaoyan815@163.com; 2 R & D Center, China Tobacco Yunnan Industrial Co., Ltd., Kunming 650231, China; wangmeng19871004@163.com (M.W.); leisheng19850928@163.com (S.L.); 3 Institute (College) of Integrative Medicine, Dalian Medical University, Dalian 116044, China

**Keywords:** bioactive-component screening, *Herba epimedii*, herbal medicine, quantitative pattern–activity relationship

## Abstract

Screening of bioactive components is important for modernization and quality control of herbal medicines, while the traditional bioassay-guided phytochemical approach is time-consuming and laborious. The presented study proposes a strategy for rapid screening of active components from herbal medicines. As a case study, the quantitative pattern–activity relationship (QPAR) between compounds and the osteoclastic inhibitory effect of *Herba epimedii*, a widely used herbal medicine in China, were investigated based on joint models. For model construction, standard mixtures data showed that the joint-action models are better than the partial least-squares (PLS) model. Then, the Good2bad value, which could reflect components’ importance based on Monte Carlo sampling, was coupled with the joint-action models for screening of active components. A compound (baohuoside I) and a component composed of compounds with retention times in the 6.9–7.9 min range were selected by our method. Their inhibition rates were higher than icariin, the key bioactive compound in *Herba epimedii*, which could inhibit osteoclast differentiation and bone resorption in a previous study. Meanwhile, the half-maximal effective concentration, namely, EC_50_ value of the selected component was 7.54 μg/mL, much smaller than that of baohuoside I—77 μg/mL—which indicated that there is synergistic action between compounds in the selected component. The results clearly show our proposed method is simple and effective in screening the most-bioactive components and compounds, as well as drug-lead components, from herbal medicines.

## 1. Introduction

The long-term clinical practice of herbal medicine confirms its importance and essential role in the healthcare system in China, especially in the prevention and treatment of chronic diseases [1,2,3]. However, the major obstacle to the development of herbal medicine is to determine the bioactive and toxic constituents in herbal medicines [4,5,6]. The bioactive constituents of the Chinese herbal medicines for a certain disease remain largely unknown, except a few examples including taxol from the Pacific yew for anticancer treatment, and artesunate from *Artemisia annua* L. for malaria treatment [7]. Bioassay-guided fractionation is the conventional phytochemical approach to screen active components from herbal medicine, but it is time-consuming and laborious [8,9,10,11]. Moreover, more than one compound or component contributes to the activity of herbal medicines, and synergistic actions always exist among different constituents [12,13]. Rapid, effective and economical approaches should be developed for screening active components from herbal medicines.

The quantitative pattern–activity relationship (QPAR) approach has been proposed in recent years, which quantitatively correlates the chromatographic fingerprint and related bioactivity capacities of the samples by partial least-squares (PLS) [12,14,15], artificial neural network (ANN) [16] and other regression methods, where the quantitative pattern is the quantitative or semi-quantitative result by HPLC–UV, HPLC–MS, NMR spectroscopy and so on. Compared with conventional bioactive-component screening approaches, QPAR has great advantages due to being fast, efficient and allowing component-group screening. Now, PLS coupled with feature selection is the favorite method in QPAR [17,18,19]. The main problem of PLS lies in that it is a linear method. However, the relationship between component concentration and activity is not a linear correlation, but a sigmoid or hyperbolic correlation [20,21,22]. Therefore, feature selection based on PLS is an under-fitting method. Although ANN is a nonlinear method, it is inclined to over-fitting and it is hard to explain the relationship between each component.

It is well known that the relationship between component dose and activity could be fitted by the Hill equation in most cases [23]. Concentration addition (CA) [24] and response addition (RA) [25] are two basic joint-action models for modeling mixture activity based on the Hill equation, which is widely used for evaluating mixture activity in the pharmaceutical industry and in environmental risk assessment [26,27]. CA is used for components with similar mechanisms/modes of action (MoA) and RA for components with dissimilar MoA. However, the mixture is more complicated in herbal medicines, which may contain components with similar and dissimilar MoA influencing the final activity together. Thus, based on CA and RA, Olmstead proposed an integrated model for modeling a mixture containing components with similar and dissimilar MoA [28]. The Olmstead model preserves the obvious advantages of the clear relationships of components and a reasonable equation expression.

In this study, three joint-action models coupled with Monte Carlo sampling (MCS) were used for screening bioactive components in *Herba epimedii* for osteoclast growth inhibition. *Herba epimedii* has been used for thousands of years to treat osteoporotic conditions. Icariin, a flavonol glycoside, is one of the primary ingredients of *Herba epimedii*. Icariin has been shown to enhance osteogenic differentiation of rat bone-marrow stromal stem cells by increasing the transcriptional co-activators TAZ expression. Meanwhile, icariin protects against glucocorticoid-induced osteoporosis, increases the expression of the bone enhancer DEC1 and modulates the PI3K/Akt/GSK3b/b-catenin integrated signaling pathway [29,30]. Active components and compounds in *Herba epimedii* selected by the Olmstead model were prepared and validated. Results indicated that the joint-action models can reveal the latent components and compounds rapidly and accurately.

## 2. Results and Discussion

### 2.1. Model Comparison

**Figure 1:**
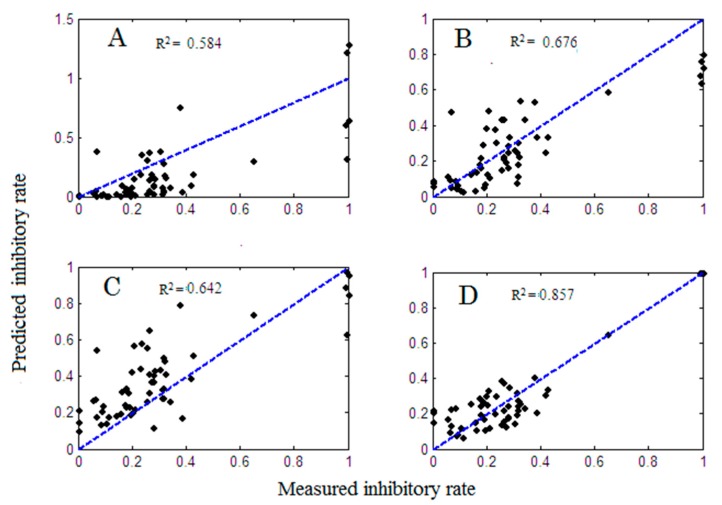
Model adequacy plot of six-standard mixtures by partial least-squares (PLS, **A**), concentration addition (CA, **B**) model, response addition (RA, **C**) model and the Olmstead model (**D**).

Table 1 lists the 5%, 10%, 20%, 30%, 40% and 50% maximal effective concentration, namely, EC_5_, EC_10_, EC_20_, EC_30_, EC_40_ and EC_50_ values of six standards, which were determined in the RAW264.7 growth-inhibition test. The final concentration ratios of six standards through uniform design and the equivalent concentration method in mixtures 1–8 are summarized in Table S1. Figure 1 shows the model adequacy plot of six-standard mixtures by the joint-action and PLS models. The *x*-axis represents the measured osteoclastic inhibitory abilities of mixtures 1–8 under seven concentrations and the *y*-axis represents the predicted activities of PLS, CA, RA and the Olmstead model. The closer the R-square values were to 1, the closer the measured activities were to the predicted activities. The R-square values modeled by PLS, CA, RA and the Olmstead model were 0.548 (Figure 1A), 0.676 (Figure 1B), 0.642 (Figure 1C) and 0.857 (Figure 1D), respectively. It showed clearly that the joint-action models, especially the Olmstead model, preserve an obvious advantage over the PLS model. Therefore, QPAR analysis based on the joint-action model may reveal a more accurate result.

### 2.2. Chromatographic Profile of Herba epimedii and Feature Extraction

**Figure 2:**
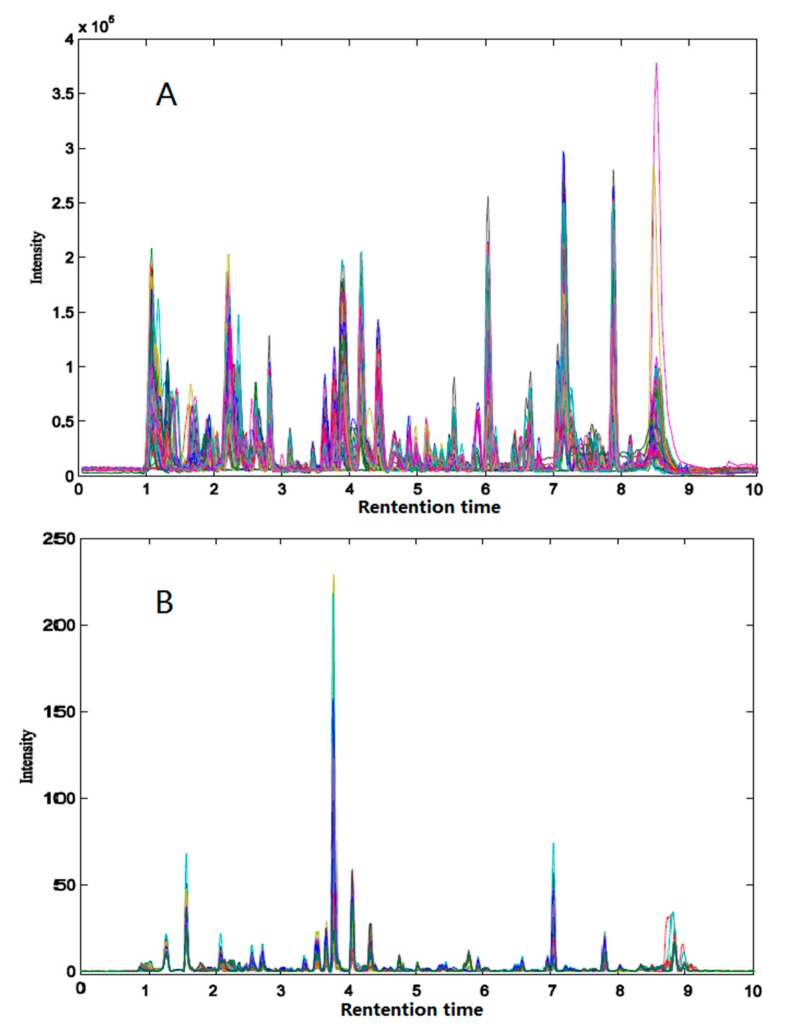
Overlapped total ion chromatograms (**A**) and liquid chromatograms (**B**) of paired samples after baseline correction and alignment by ChromP. The chromatograms of different paired samples show different colors in the diagram.

Figure 2 shows the overlapped total ion chromatograms (TICs, Figure 2A) and liquid chromatogram diode array detector (DAD) data (Figure 2B) of 66 *Herba epimedii* paired samples after baseline correction and alignment by ChromP. The preparation method of 66 pairwise samples is described in Section 3.2.1. The theoretical basis of QPAR lies in the fact that the varied quantity of bioactive compounds will influence the activity results of the herbal samples. As a result, component content variance is necessary for the QPAR study. For paired samples, the coefficient of variation (CV), namely, the relative standard deviation (RSD) of the TIC and DAD of compounds in all retention times, are shown in Supplementary Materials Figure S1. For the TIC, the CVs of compounds’ area in all samples varied from 0.119 to 1.323, as shown in Figure S1A, indicating a useful variation for model construction. For DAD data, the CVs of most of the compounds’ areas in all samples were less than 10, although some compounds’ areas reached 20, as shown in Figure S1B. By using ChromP, there were totals of 73 and 61 features extracted from the DAD and TIC, respectively. Compared with XCMS and MZmine, ChromP tends to extract the visual and unduplicated features just from the chromatogram. Thus, it is a more-suitable global search method.

### 2.3. Similarity Analysis of Extracted Features

**Figure 3:**
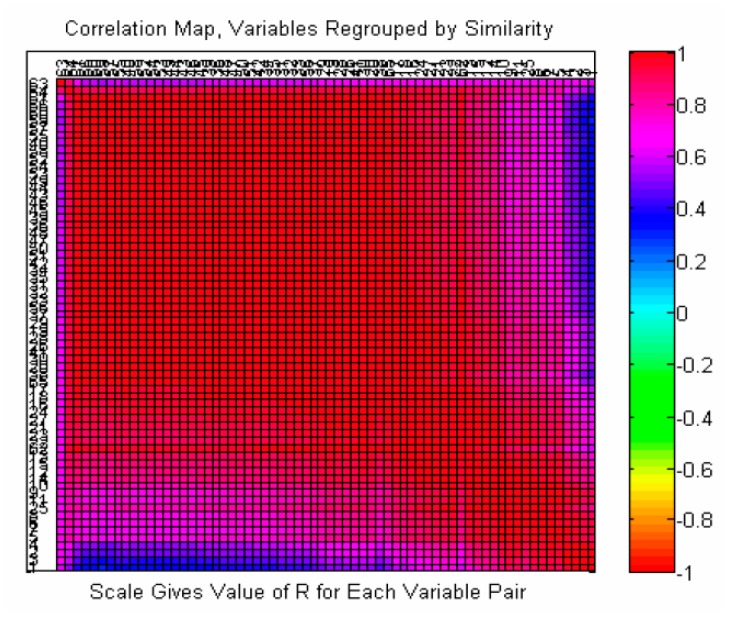
UV spectrum correlation heat-map of 73 features extracted from diode array detector (DAD) data.

The overlapped UV spectrum from 200 to 400 nm of each feature is shown in Figure S2. Features extracted from the TICs and liquid chromatograms were integrated together, so that all features belonging to the TICs were also included within the liquid chromatograms. In Figure S2, it is obvious that some features exhibit similar UV spectra, indicating that they could be grouped in a cassette in the Olmstead model. Figure 3 displays a UV spectrum correlation heat-map of 73 features extracted from DAD data. Most of the features have a correlation coefficient of more than 0.9, as shown in red in Figure 1, which should be clustered into one group. The features with correlation coefficients between 0.6–0.8 and 0.3–0.5, marked with purple and blue in Figure 3, respectively, should be clustered into another two groups. Apparently, 73 features should be clustered into three groups. Most of those compounds show strong correlations near to 1. This is reasonable for those compounds from species tending to share the same basic structure. At the same time, a small number of compounds display strong correlations with each other but poor correlations with others, which may indicate another category of component. Figure S3 shows an additional proof of compound grouping: principle component analysis (PCA) scatter plot of the UV spectrum of 73 features. By the k-means algorithm, 73 features were tentatively divided into categories one, two, three and four. The results showed that compounds should be clustered into three categories, according to the UV spectra.

### 2.4. Inhibition Activity to RAW264.7

**Figure 4:**
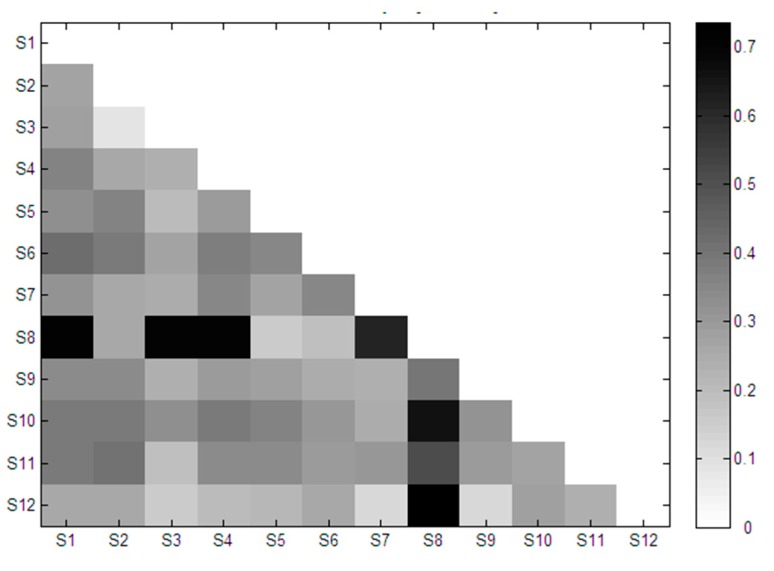
The heat-map of osteoclastic inhibitory activities of paired *Herba epimedii* samples. S1–S12, mean sample 1–sample 12 of *Herba epimedii*.

The activity heat-map of paired *Herba epimedii* samples are shown in Figure 4. The activity of different paired samples varied greatly. The maximum, minimum, mean and standard deviation of activity was 0.74, 0.09, 0.33 and 0.14, respectively. The different activities lie in the variability of bioactive-component quantity in different paired samples, which is the basis of QPAR. 

### 2.5. Results of Data Processing

**Figure 5:**
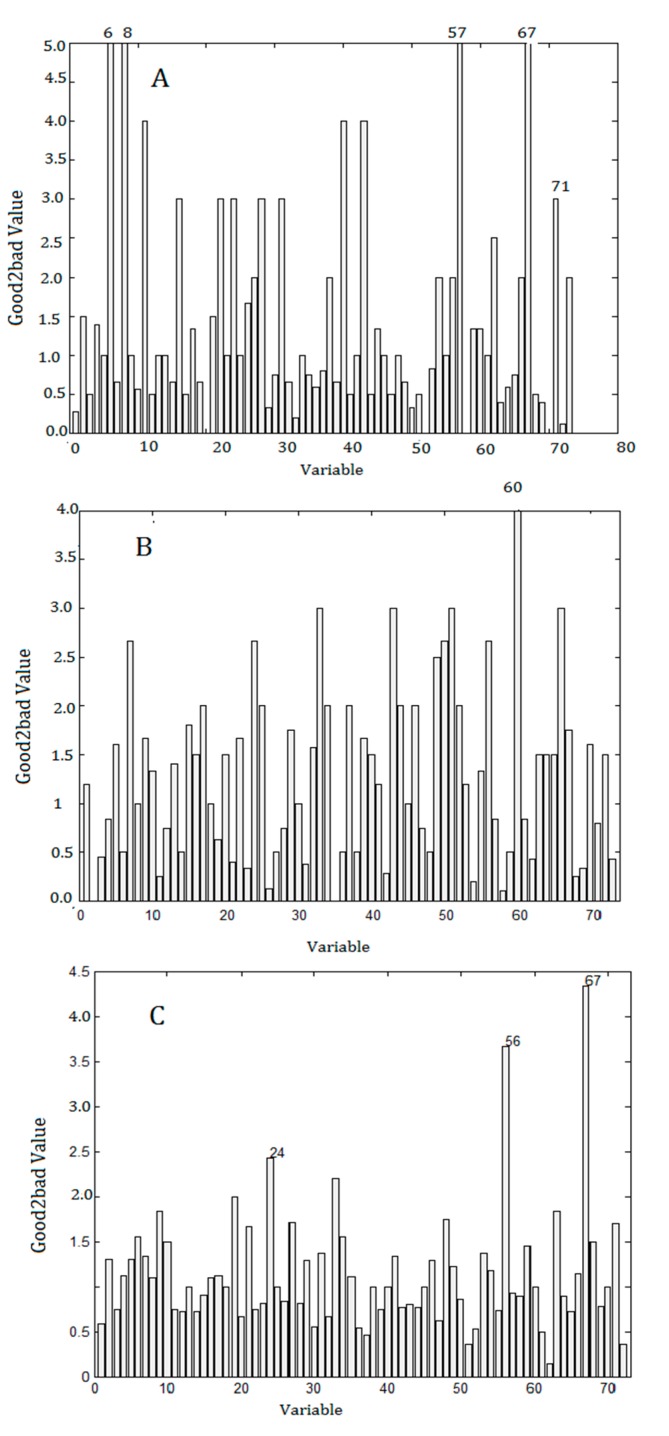
Good2bad values performed on the liquid chromatogram data through concentration additionmodel (CA, **A**), response addition model (RA, **B**) and the Olmstead model (**C**).

The Good2bad analysis was performed on the liquid chromatogram data through CA, RA and the Olmstead models. Figure 5 shows the selected Good2bad values under different models. Generally, the top 1–3 features were selected as latent bioactive compounds. Table 2 lists the selected latent bioactive compounds by different models. In Table 2, the retention time (RT) of compound **56** and **67** selected by the Olmstead model was 6.57 min and 7.9 min, respectively. Compound **56** was identified as sagittatoside A and compound **67** was identified as baohuoside I [31]. The activity of sagittatoside A was validated by Zhang et al. [32]. Baohuoside I was simultaneously screened by the CA and the Olmstead models. Meanwhile, it was also screened by the controlled PLS model. Results indicated baohuoside I may be a latent bioactive compound. The retention times of most of the selected compounds were around 6.9–7.9 min, which indicated that these components may be latent bioactives.

### 2.6. Validation of Selected Components and Compounds

**Figure 6:**
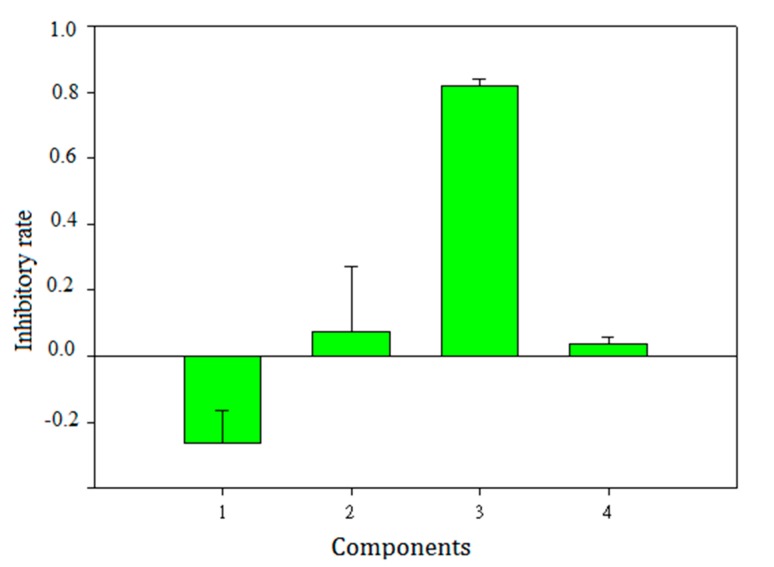
Inhibitory rates of different components **1**, **2**, **3** and **4** to RAW264.7 growth. Components **1**, **2**, **3** and **4** were obtained by separating the extract of *Herba epimedii* on a HPD resin column eluting with 10%, 50%, 80% and 100% ethanol, respectively.

**Figure 7:**
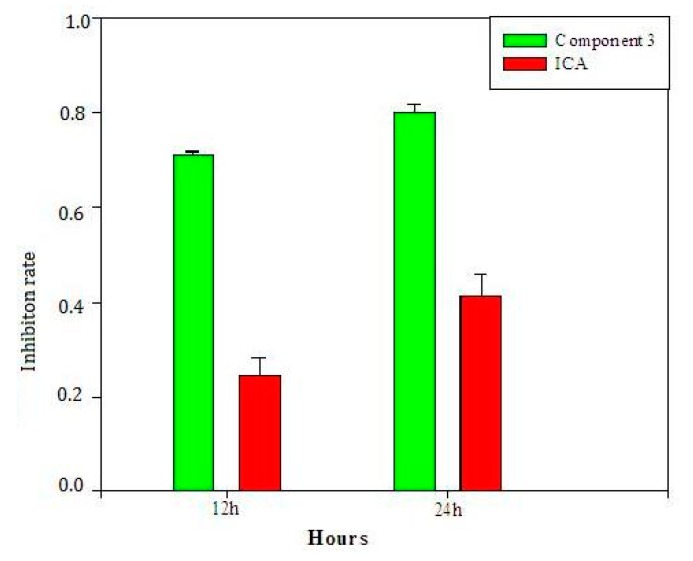
Comparison of activities of component **3** and icariin after 12 and 24 h incubation.

**Figure 8:**
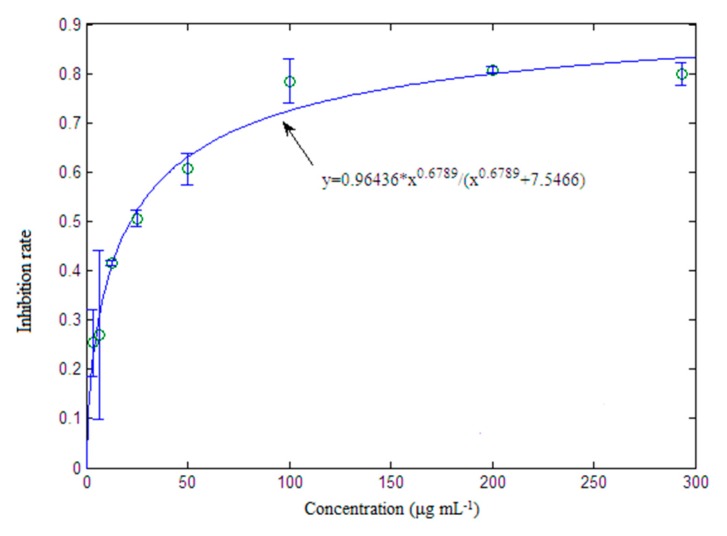
Concentration-response curve (CRC) of component **3** and its inhibition of RAW264.7 growth.

The latent bioactive components and compounds were validated by the cell assay depicted in Section 3.4. Components **1**, **2**, **3** and **4** were obtained from the extract of *Herba epimedii* on a HPD resin column as depicted in Section 3.6.5. Component **3** included most of the selected compounds with retention times around 6.9–7.9 min, which indicated that component **3** may be a latent bioactive component. Figure 6 is the RAW264.7 inhibition rate of the four components. It shows clearly that component **3** has an activity far superior to that of the other components. The inhibition rate of component **3** was almost 81.2%, while the inhibition rates of other components were below 7%. This indicated that compounds in component **3** may be the main bioactive chemicals in *Herba epimedii*. Meanwhile, the activity of component **3** was also higher than that of icariin, the key bioactive chemical in *Herba epimedii* that could inhibit osteoclast differentiation and bone resorption in a previous study [33] after 12 and 24 h incubation, respectively (Figure 7). Figure 8 shows the concentration-response curve (CRC) of component **3**’s inhibition of RAW264.7 growth, where its EC_50_ value is 7.54 μg/mL. 

Activities of baohuoside I and icariin have been compared in our previous work [34], where the maximum activity of baohuoside I could reach 91% and icariin reached 60%. Meanwhile, in the osteogenic differentiation test, induced nitric oxide synthase (iNOS) and nitric oxide (NO) production were greatly promoted after adding baohuoside I [35]. All these data indicate that baohuoside I preserves higher activity for osteoclast growth inhibition than icariin.

By comparison of component **3** with baohuoside I, although the maximum activity of baohuoside I was larger than component **3**, the EC_50_ value of component **3** was 7.54 μg/mL, much lower than that of baohuoside I, 77 μg/mL. This indicated that there may be a synergistic effect of compound combinations, such as baohuoside I, sagittatoside A, 2′′-*O*-rhamnosyl icariside II, or other unidentified compounds in component **3** [36]. 

## 3. Materials and Methods

### 3.1. Reagents and Materials

HPLC-grade acetonitrile and 3-(4,5-dimethylthiazol-2-yl)-2,5-diphenyltetrazolium bromide were purchased from Sigma-Aldrich (St. Louis, MO, USA). HPLC-grade water was prepared using a Milli-Q water purification system (Millipore, Boston, MA, USA). The mouse macrophage pre-osteoclastic RAW264.7 cells were obtained from the Chinese Academy of Sciences (Shanghai, China). Standards of the compounds epimedin A (**1**), epimedin B (**2**), epimedin C (**3**), icariin (**4**), baohuoside I (**5**) and icartin (**6**) were isolated in our laboratory (purity > 98% based on the peak areas of HPLC in this assay). Their structures were unequivocally identified based on UV, IR and NMR spectroscopic analysis. Twelve *Herba epimedii* leaf samples were purchased from Zhi Lin Large Pharmacy Chain Co. Ltd in different locations of China and were identified by Dr. Yujing Zhang from the Department of Pharmacognosy of the School of Pharmacy, Zunyi Medical University (Zunyi, China). Table S2 lists the detailed locations of each pharmacy. The samples were dried in an oven for 2 h before use.

### 3.2. Sample Preparation

#### 3.2.1. Preparation of Standard Mixtures for Model Comparisons

For preparation of standard mixtures, a procedure was introduced as below: firstly, six standards were weighed accurately and dissolved in dimethyl sulfoxide (DMSO) to prepare stock solutions with concentrations of 400 μg/mL. The prepared standards solutions were diluted two times step by step to get seven concentrations. The EC_5_, EC_10_, EC_20_, EC_30_, EC_40_ andEC_50_ values of each standard were calculated by the Hill equation according to the osteoclastic inhibitory abilities of standards 1–6 described in Section 3.4. Secondly, we composed mixtures 1–6 of six standards through uniform design [37]. A uniform design table of U_7_(7^6^) (six factors and seven levels, Table S3) was used for composition of mixtures 1–6, where the standards indicate factors and EC_5_–EC_50_ of each standard indicate levels. Thus, the U_7_(7^6^) table is sufficient for a six-factors and seven-levels test. Then, we composed mixtures 7–8 of six standards through the equivalent-effect method [38]. Equivalent-effect means the concentration needed when an equivalent effect was elicited. In our test, EC_50_ and EC_5_ were used for mixtures 7 and 8, respectively. Finally, as mentioned above, the composition design of six standards in mixtures 1–8 was established (Table 3). According to the EC_5_ to EC_50_ values of each standard, the composition ratio of each standard in every mixture is available. Take ratio of standard 1 in mixture 1 as an example, which could be expressed as follows: ratio of standard 1 = Standard 1(EC_5_)/([Standard 1(EC_5_) + Standard 2(EC_10_) + Standard 3(EC_20_) + Standard 4(EC_30_) + Standard 5(EC_40_) + Standard 6(EC_50_)]). The total concentration of all standards in each mixture is 500 μg/mL. Then, the prepared solutions of mixtures 1–8 were diluted two times step by step to get seven concentrations, and their osteoclastic inhibitory abilities were also tested, as Section 3.4 depicted.

#### 3.2.2. Preparation of *Herba epimedii* Pairwise Samples

Twelve dried *Herba epimedii* leaf samples mentioned in Section 3.1 were ground into a fine powder using a pulverizer prior to extraction. An amount of 2.0 g of each sample was extracted ultrasonically with 20 mL 80% ethanol twice for 20 min. The extract was combined and filtered, then condensed and purified by HPD macro-porous absorbent resin column chromatography to remove pigments and strong hydrophilic components. The eluate was condensed by evaporation under reduced pressure and freeze-dried to get dry powder for pairwise sample preparation.

2 mg of powder was weighed accurately and dissolved in 4 mL of DMSO in an ultrasonic bath to get a block solution. Pairwise samples were obtained by a fixed ratio of 1:1 of each block solution at 100 μL of aliquot to get component variations. Thus, 66 paired extract samples were gained for subsequent chromatographic analysis and cell activity tests.

### 3.3. Apparatus and Analytical Conditions

#### 3.3.1. Chromatographic Analysis

All analyses were carried out on an Agilent Q-TOF 6520 mass spectrometer (Agilent, Santa Clara, CA, USA) equipped with a DAD detector, autosampler and column compartment. Chromatographic separations were performed on a Agilent Proshell 120 SB-C18 column (3.0 mm × 150 mm, 2.7 μm) by gradient elution using a mobile phase consisting of HPLC-grade acetonitrile (solvent A) and ultrapure water (solvent B). The gradient elution was carried out as follows: 0–7.0 min, 25–55% A; 7.0–7.1 min, 55–100% A and 100% of solvent A holding for 2.9 min. The flow rate was kept at 0.5 mL/min. The column temperature was set to 45 °C and UV measurements were obtained at 267 nm. An aliquot of 2 μL of each sample block solution was injected onto the UPLC. Samples were filtered with 0.22 nm filter membrane prior to chromatographic analysis.

#### 3.3.2. Q-TOF/MS Analysis

MS detection was performed using an Agilent Q-TOF 6520 mass spectrometer (Agilent, Santa Clara, CA, USA) equipped with an electrospray ionization (ESI) interface. The operating parameters were as follows: drying gas (N_2_) flow rate, 8.0 L min^−1^; drying gas temperature, 320 °C; nebulizer, 45 psig; vaporizer temperature, 350 °C; capillary voltage, 3500 V; corona current, 20 μA; skimmer voltage, 65 V; fragmenter voltage, 175 V. The system was operated under MassHunter Workstation software version B.0 2.00 (Agilent, Santa Clara, CA, USA). Each sample was analyzed in negative ion mode, and the spectra were recorded in the *m*/*z* range of 100–1500 for full-scan MS analysis. 

### 3.4. Activity Test

The mouse macrophage pre-osteoclastic RAW264.7 cells were cultured in Dulbecco’s Modified Eagle’s Medium (DMEM). The RAW264.7 cells in exponential phase were seeded on 96-well plates (1 × 10^5^ cells/well) and *Herba epimedii* paired extract samples were added to the plates at a final concentration of 100 μg/mL. The plates were incubated further for 24 h in a CO_2_ incubator at 37 °C. Activity of inhibition to RAW264.7 cells was accessed by the 3-(4,5-dimethylthiazol-2-yl)-2,5-diphenyltetrazolium bromide (MTT, Sigma, St. Louis, MO, USA) assay. Inhibition rate was estimated by the following equation:

(1) $$I=\left(A_{t}-A_{b}\right)/\left(A_{c}-A_{b}\right)$$

where *I*, *A_t_*, *A_b_* and *A_c_* are inhibition rate, absorption of test sample, background (containing only DMEM) and control group (without drug) at 490 nm, respectively.

### 3.5. Theoretical Basis of Data Analysis

#### 3.5.1. Joint-Action Models

For the QPAR study, the model- and feature-selection methods are two important factors when deciding the selection results, where the model links quantitative patterns and biological activity, and the feature-selection method is concerned with selecting subsets of relevant features.

(2) $$\mathrm{CA}\;\mathrm{model}:{\mathrm{EC}}_{x,\mathrm{mix}}={\left(1+\frac{1}{{\left(\sum_{i=1}^{x}\;\frac{c_{i}}{{\mathrm{EC}}_{50i}}\right)}^{p}}\right)}^{-1},$$

where *EC*_*x*,*mix*_ is the effect of a mixture containing *x* chemicals, *c_i_* is the concentration of chemical *i*, *EC50_i_* is the concentration of chemical *i* eliciting a half-maximal response, and *p* is the exponent of chemical 1 to *x*.

(3) $$\text{RA model}:{\mathrm{EC}}_{x,\mathrm{mix}}=1-\prod_{i=1}^{x}(1-{\mathrm{EC}}_{i}),$$

where *EC*_*x*,*mix*_ also means the effect of a mixture containing *x* chemicals and *EC_i_* is the effect that chemical *i* elicits alone. 

The Olmstead model is displayed below:

(4) $${\mathrm{EC}}_{x,\mathrm{mix}}=1-\prod_{i=1}^{m}{\left(1-\frac{1}{1+{\left(\sum_{i=1}^{x}\;\frac{c_{i}}{{\mathrm{EC}}_{50i}}\right)}^{P_{\arg,i}}}\right)}^{},$$

where *EC*_*x*,*mix*_ also means the effect of a mixture containing *x* chemicals, *P_arg_,_i_* is the average power of chemicals 1 to *m* (cassette *m*), which reflects the important index of cassette *m* to some extent, *c_i_* is the concentration of the *i^th^* chemical and *EC*_50*i*_ is the half-maximal effective concentration of *i^th^* chemicals when singly used. 

The Olmstead model is the combination of CA and RA models mentioned above. In this model, compounds with similar MoA are firstly grouped in a cassette as the CA model, and then compounds in different cassettes are combined as the RA model. Cassette number is a key factor for the joint-action model. When cassette number is 1, the Olmstead model is indeed a CA model. When cassette number is equal to compound number in the mixture, the Olmstead model is indeed an RA model. Cassette number is concerned with MoA similarity of each compound in the mixture. However, for herbal medicines, it is hard to determine the MoA of each constituent chemical. Therefore, for the Olmsted model, cassette number and compound cassette attribution should be decided first.

#### 3.5.2. Cassette-Number Evaluation of the Olmstead Model

Absorption spectra could reflect a chemical structure to some extent [39]. The UV spectrum of a compound is related to the electromagnetic spectrum and electronic-transition information, and compounds with certain UV-wavelength absorption represent certain structural groups. If compounds have a similar structure, they will display similar MoA and UV spectra. 

By UPLC coupled with a DAD or PDA detector, the UV spectrum of each compound in the mixture is available. The compounds are then grouped according to their UV spectrum by hierarchical clustering [40] and similarity analysis. Thus, the cassette information of the Olmstead model is established.

#### 3.5.3. Good2bad Value Analysis Based on Monte Carlo Sampling

The main concept of Monte Carlo sampling (MCS) lies in it randomly and iteratively drawing a great number of subsets of the sample and feature direction together to construct *N* sub-models [41]. For each sub-model, interesting parameters are calculated for further analysis. The Good2bad value is proposed for selecting relevant variables based on MCS.

Figure S4 reflects the principle of the Good2bad value. Its main theory and calculation steps are listed below: *N* models were firstly constructed based on *N* subset after MCS, and the model error of each Olmstead model was calculated. Generally, prediction error and recognition error have a normal distribution. For a normal distribution error, the left tail part indicates models with small error (SEM), while the right tail part indicates models with large error (BEM), as shown in Figure S4A. A certain fraction, such as 1% or 5%, could be used to count SEM and BEM quantitatively, according to the balance of computational tolerance and variable selective frequency. Then, variable frequency in SEM and BEM were calculated, where the variable presents a high frequency in SEM indicating importance to the Olmstead model construction, and the opposite situation in BEM (Figure S4B,C). The variable importance could be expressed as the follow equation:

Good2bad (*i*) = F(*i*)SEM/F(*i*)BEM, (5)

where F(*i*)SEM represents selected frequency of variable *i* in SEM, the same as F(*i*)BEM. The bigger the F(*i*)SEM and the smaller the F(*i*)BEM is, the more important variable *i* is.

Thus, as shown in Figure S4D, compounds with larger Good2bad values, such as variables 1, 2, 4 and 5, would be classified as relevant variables; on the other hand, variable 3, with a much smaller Good2bad value, indicates an irrelevant variable [42]. 

### 3.6. Bioactive-Component Screening Procedure and Parameter-Setting

#### 3.6.1. Feature Extraction from Chromatographic Profile

XCMS [43], MZmine [44] or ChromP is used for signal deconvolution, noise filter, peak alignment and feature extraction. After feature extraction, a data matrix containing samples and features (chemical information) is available.

#### 3.6.2. Obtain UV Spectrum of Each Compound and Cluster Analysis

The UV spectrum of each compound was extracted according to the retention time of extracted features. K-means was adopted for cluster analysis. The cassette of each compound, that is, the group number, was decided according to feature distribution in the principle component analysis (PCA) score plot.

#### 3.6.3. Construct Quantitative Pattern–Activity Model Based on Subsets

Subsets were generated by MCS. The detailed parameter settings of MCS in our test were: iteration 1000; number of variable 0.05; number of sample 0.7. Each subset was modeled by the joint-action models, and parameters were estimated by the global search method [45]. Samples were selected in training and tested randomly. The cassette information of each compound was decided by the cluster analysis result. All the data were standardized by min–max normalization before modeling. For comparison, different groups and PLS methods were also used for bioactive-component selection.

#### 3.6.4. Good2bad Analysis

After MCS, model errors of all subsets were analyzed, and Good2bad values for each feature were computed to reflect the importance of variables. 

#### 3.6.5. Verification

The selected latent bioactive components and compounds were prepared and verified by biological assay as depicted in Section 3.4. For latent active component, the extract of *Herba epimedii* was sampled on an HPD resin column, and eluted by 10%, 50%, 80% and 100% ethanol, and components **1**, **2**, **3** and **4** were obtained after condensation. The component **3** contained the latent active component by UPLC analysis. For preparation of the latent compound, an enzyme-hydrolysis method was established in our previous work [46]. Generally, the latent compound was prepared by dextranase hydrolyzing icariin at pH of 5.4, temperature of 40 °C and hydrolysis time of 3 h.

## 4. Conclusions

The currently used traditional Chinese medicine (TCM) (herbal medicine) is mostly prepared by a simple extraction, which contains hundreds of useless, unknown or even toxic components. Now, TCM simplification, also known as TCM redevelopment, is a key project for TCM modernization. For TCM simplification, the crucial problem is to decide which regions are bioactive, inactive or toxins in the chromatographic profile and the possible synergistic effect of compound combinations. Then, a directed preparation is needed. 

By the QPAR method, the effective bioactive components could be revealed, giving an opportunity for TCM simplification. A QPAR method based on joint-action models was developed for screening bioactive components with RAW264.7 growth-inhibition activity in *Herba epimedii*. By combining the screening results of TIC and DAD quantitative data, a bioactive component and a compound (baohuoside I) were selected from *Herba epimedii*. As expected, their activity was superior to icariin, a key bioactive compound in *Herba epimedii* that exhibits an osteoclastic inhibitory effect. The EC_50_ value of the selected component is smaller than that of the selected compound (baohuoside I). Baohuoside I with retention time of 7.9 min was included in the selected component. Therefore, there is a synergistic effect between compounds in the selected component. The results indicated that the joint-action model is more powerful than the PLS model in bioactive-component screening. The presented study proposes a strategy for rapid screening of active candidates from herbal medicines.

## Figures and Tables

**Table 1:** EC_5–50_ *^a^* values of six standards in the RAW264.7 growth inhibition test.

Standards	Name	EC_5_	EC_10_	EC_20_	EC_30_	EC_40_	EC_50_
**1**	epimedin A	8.11	41.00	238.09	766.50	1998.72	4816.47
**2**	epimedin B	7.14	19.86	60.32	126.22	231.21	402.92
**3**	epimedin C	0.02	0.52	18.53	199.07	1394.17	8318.58
**4**	icariin	0.91	3.24	12.80	31.91	67.48	134.17
**5**	baohuoside I	13.77	21.32	34.26	46.95	60.79	77.06
**6**	icartin	1.90	4.73	12.73	24.56	42.09	69.02

*^a^* EC5, EC_10_, EC_20_, EC_30_, EC_40_ and EC_50_ mean the 5%, 10%, 20%, 30%, 40% and 50% maximal effective concentration of standards.

**Table 2:** DAD and TIC data of selected compounds by different models.

No. *^a^*	M^−^	RT (min)	Data Source	Model	Compounds
6	448.1861	1.68	DAD	CA	NA *^b^*
8	609.0456	1.80	DAD	CA	NA
11	564.4197, 627.4082	2.39	TIC	RA/CA	NA
33	661.2183, 724.2124	5.12	TIC	RA	Icarisoside B
50	659.2383, 722.2349	7.16	TIC	RA	2″-*O*-Rhamnosyl icariside II
56	675.2338, 738.2297	6.94	DAD	Olmstead	Sagittatoside A
57	659.2383, 722.2349	7.04	DAD	CA	2″-*O*-Rhamnosyl icariside II
60	717.2413, 780.2374	7.50	DAD	RA	NA
67	513.1794, 576.1794	7.90	DAD	Olmsted/CA/PLS	Baohuoside-I

*^a^* Number of selected features from 73 features extracted from DAD; *^b^* NA means unknown compound.

**Table 3:** Mixing ratio of six standards in each mixture.

Mixtures *^b^*	Standards (μg/mL) *^a^*					
	1	2	3	4	5	6
1	EC_5_	EC_10_	EC_20_	EC_30_	EC_40_	EC_50_
2	EC_10_	EC_30_	EC_50_	EC_5_	EC_20_	EC_40_
3	EC_20_	EC_50_	EC_10_	EC_40_	EC_5_	EC_30_
4	EC_30_	EC_5_	EC_40_	EC_10_	EC_50_	EC_20_
5	EC_40_	EC_20_	EC_5_	EC_50_	EC_30_	EC_10_
6	EC_50_	EC_40_	EC_30_	EC_20_	EC_10_	EC_5_
7	EC_50_	EC_50_	EC_50_	EC_50_	EC_50_	EC_50_
8	EC_5_	EC_5_	EC_5_	EC_5_	EC_5_	EC_5_

*^a^* Standards **1**, **2**, **3**, **4**, **5** and **6** were epimedin A, epimedin B, epimedin C, icariin, baohuoside I and icartin, respectively; *^b^* mixtures 1–6 were composed of fixed concentration ratios of six standards through uniform design, and mixtures 7 and 8 were composed of fixed concentration ratios of six standards through the equivalent concentration method.

## Supplementary Materials

The following are available online. Figure S1: CV of compounds’ area in all *Herba epimedii* samples in total ions chromatogram (TIC, A) and liquid chromatogram (B). Figure S2: Overlapped UV spectrum of 73 compounds extracted from liquid chromatogram. Figure S3: Principle component analysis (PCA) score plot of UV spectrum of 73 features, Table S1: Final concentrations of 6 standards in mixtures 1–8, Table S2: Information of *Herba epimedii* leaf samples purchased in experiment.
