# Supplementary material for: Rapid Screening of Active Components with an Osteoclastic Inhibitory Effect in Herba epimedii Using Quantitative Pattern–Activity Relationships Based on Joint-Action Models
Source: Molecules. 2017 Oct 19;22(10):1767. doi: 10.3390/molecules22101767 (PMC6151442; doi:10.3390/molecules22101767)
Supplement: Supplementary file 1 [file molecules-22-01767-s001.pdf]

Supplementary Materials:

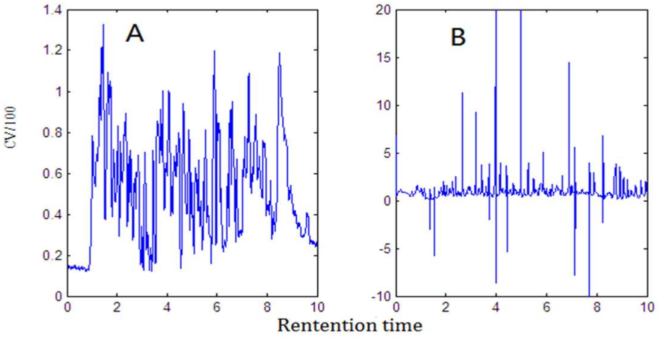

Figure S1. CV (RSD) of compounds' area in all *Herba Epimedii* samples in total ions chromatogram (TIC, A) and liquid chromatogram (B).

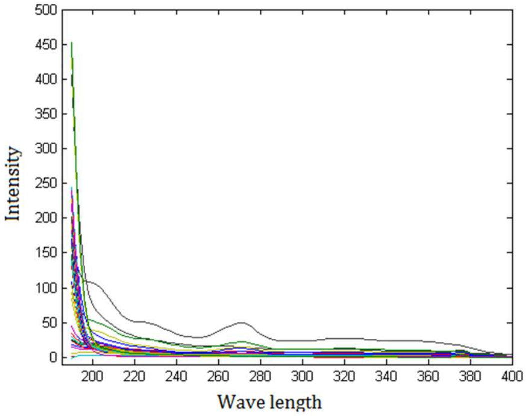

Figure S2. Overlapped UV spectrum of 73 compounds extracted from liquid chromatogram.

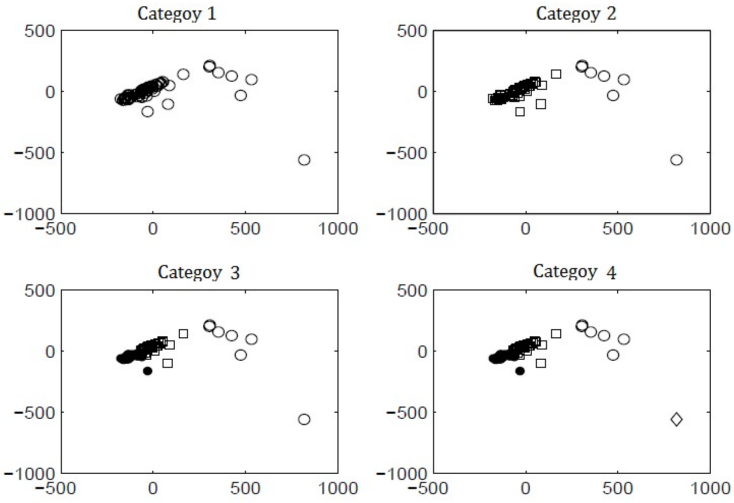

Figure S3. Principle component analysis (PCA) score plot of UV spectrum of 73 features.

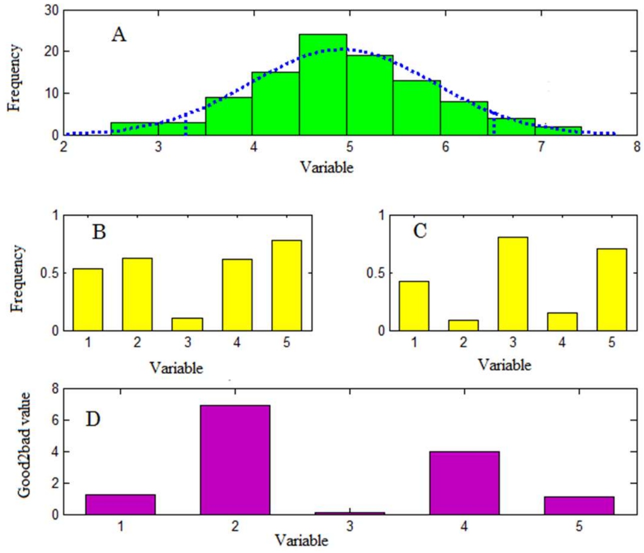

Figure S4. Principle of Good2Bad value based on Monte Carlo Sampling: normal distribution sub-models errors (A), variable frequency of models with small error (B), variable frequency of models with big error (C) and Good2bad values (D).

Table S1. Final concentrations of 6 standards in mixtures 1-8.

Mixtures	Standards/μg/mL					
	**1**	**2**	**3**	**4**	**5**	**6**
1	19.46	47.69	44.48	76.63	145.98	165.73
2	2.39	7.37	485.72	0.05	2.00	2.45
3	159.29	269.56	0.34	45.14	9.21	16.43
4	169.51	1.57	308.33	0.71	17.04	2.81
5	445.16	13.43	0.01	29.88	10.45	1.05
6	455.86	21.88	18.84	1.21	2.01	0.18
7	174.27	14.57	301.00	4.85	2.78	2.49
8	127.26	112.03	0.30	14.32	216.20	29.87

Table S2. Information of *Herba Epimedii* leaf samples purchased in experiment.

Samples	Taxon	Source
1	Epimedium koreanum Nakai	Haerbin, Heilongjang, China
2	Epimedium brevicornu Maxim	Lingzhou, Shanxi, China
3	Epimedium koreanum Nakai	Dalian, LiaoNing, China
4	Epimedium pubescens Maxim	Bazhong, SiChuan, China
5	Epimedium brevicornu Maxim	Minxian, GanSu, China
6	Epimedium sagittatum (Sieb. et Zucc.) Maxim.	Quanzhou, GuangXi, China
7	Epimedium acuminatum Franch	Zhunyi, GuiZhou, China
8	Epimedium baojingense Q.L. Chen and B.M. Yang	Zhuzhou, HuNan, China
9	Epimedium sagittatum (Sieb. et Zucc.) Maxim.	Yunshan, AnHui, China
10	Epimedium wushanense T.S. Ying	Cangzhou, HeBei, China
11	Epimedium lishihchenii Stearn	Lushan, JiangXi, China
12	Epimedium koreanum Nakai	Baotou, NeiMeng, China

Table S3. Uniform design table of $U_7(7^6)$.

**Levels**	**Factors**					
	Factor 1	Factor 2	Factor 3	Factor 4	Factor 5	Factor 6
1	1	2	3	4	5	6
2	2	4	6	1	3	5
3	3	6	2	5	1	4
4	4	1	5	2	6	3
5	5	3	1	6	4	2
6	6	5	4	3	2	1
7	7	7	7	9	7	7
